# Myocardial deformation indices for detection of the functional significance of intermediate left anterior descending coronary artery stenosis: FFR guided study

**DOI:** 10.1007/s10554-022-02668-y

**Published:** 2022-07-12

**Authors:** Ahmed Shawky Shereef, Suaad Abdallah Ali Mosbah, Salwa Mohamed Ghoniem, Islam Elsayed Shehata

**Affiliations:** 1 Department of Cardiology, Faculty of Medicine, Zagazig University, Zagazig, 44519 Egypt; 2 College of Applied Medical Sciences, Imam Abdulrahman Bin Faisal University, Dammam, 1982 Saudi Arabia

**Keywords:** Coronary stenosis, Echocardiography, Fractional flow reserve, Speckle tracking imaging

## Abstract

This study aimed to investigate the diagnostic performance of non-invasive resting myocardial deformation indices in identifying functional significance of intermediate stenosis of the left anterior descending (LAD) artery. Patients with 50–70% LAD stenosis upon coronary angiography were enrolled and divided into group I with fractional flow reserve (FFR) > 0.8 and group II with FFR ≤ 0.8. Patients were subjected to conventional and speckle tracking echocardiography with measurement of myocardial deformation indices including regional peak longitudinal strain (PLS), global longitudinal strain (GLS), Post-systolic strain index (PSI), and time interval between Aortic valve closure (AVC) and PLS. The current study included 200 patients. Group II patients had significantly lower absolute mean values of regional (PLS) and (GLS) compared to group I (− 14.98 ± 5.05 and − 18.73 ± 3.92 vs. − 17.59 ± 3.62 and − 19.20 ± 2.61, p = 0.001 and 0.02, respectively). The FFR values of LAD correlated significantly and negatively with the time interval between AVC and regional PLS (r = − 0.201, p = 0.004) as well as PSI (r = − 0.257, p < 0.001). For identifying cases with FFR ≤ 0.8, the optimal cut-off value of the time interval between AVC and PLS was 76 ms with 77.8% sensitivity and 93.8% specificity. The best cut-off value of PSI was 13%, yielding 50% sensitivity and 87.5% specificity. In patients with intermediate 50–70% LAD coronary artery stenotic lesions, the PSI and the duration between AVC and regional PLS enabled the identification of functionally significant lesions with reasonable diagnostic accuracy.

*Trial registration* ZU-IRB#3199-20-11-2015 Registered 20 November 2015, IRB_123@medicine.zu.edu.eg.

## Introduction

Coronary angiography has been considered as a standard for assessment of coronary artery stenosis inspite of having few restrictions particularly in assessment of functional significance of intermediate stenotic lesions [1]. The accurate estimation of hemodynamic significance of these intermediate coronary lesions is challenging despite it is crucial for determination stenotic lesions that may benefit from revascularization [2, 3].

Currently, the gold standard for diagnosing hemodynamically significant intermediate stenoses in CAD patients is the fractional flow reserve (FFR). An FFR equals or less than 0.80 is used as a cut-off for identifying significant stenosis that will benefit if treated by revascularization. [3–6].

The new non-invasive measurement of myocardial deformation using speckle tracking echocardiography (STE) provides an objective reproducible evaluation of regional systolic left ventricular function and hence detection of myocardial ischemia [7].

Longitudinal strain (LS) as a myocardial deformation parameter was found to predict subtle concealed ischemia in asymptomatic patient even in absence of regional wall motion abnormalities [8]. Moreover, markers of post-systolic shortening (PSS) including the post-systolic strain index (PSI) and the duration between aortic valve closure (AVC) to regional peak longitudinal strain (PLS) can also be used to identify LV ischemic segments [9].

Therefore, this study was conducted to explore the clinical application of non-invasive resting myocardial deformation indices (regional LS, PSI, and the duration between AVC and regional PLS) in predicting the functional severity of intermediate left anterior descending (LAD) coronary artery stenosis.

## Material and methods

### Ethical considerations

The current study protocol has been evaluated and approved by our university's Institutional Review Board (IRB). The IRB guaranteed that all methods were carried out in conformity with the 1975 Declaration of Helsinki's ethical norms for human research. Informed written consent was obtained from all participants.

### Study design, settings, and date

This cross-sectional study was conducted at cardiology department of our university between June, 2016 and May, 2021.

### Sample size calculation

The sample size was calculated using MedCalc Statistical Software version 15.8 (MedCalc Software bvba, Ostend, Belgium; https://www.medcalc.org; 2015). Assuming an area under the receiver operating characteristic curve (AUC) of at least 0.7, type I error of 0.01, 90% power of the test, and a ratio of cases with FFR > 0.8 to FFR ≤ 0.8 of 2.5 based on the prevalence of 29% of FFR ≤ 0.8 that was reported by Hoole et al. [10], the calculated sample size was 151 patients (42 positive cases and 108 negative cases). Adding 20% to compensate for the loss during follow-up, the final minimal sample size was 180 (50 positive cases and 130 negative cases).

### Eligibility criteria

We enrolled consecutive patients who have functionally suspicious intermediate LAD coronary lesion (50–70% stenosis by coronary angiography) [11]. Then, the patients were divided into two groups based on FFR values of 0.8 that differentiated the functionally significant from the non-significant stenotic lesions [12]. Group I included patients with LAD intermediate stenotic lesions and FFR values > 0.8, whereas group II included patients with LAD intermediate stenotic lesions and FFR values ≤ 0.8.

Patients with significant lesions other than LAD, a history of myocardial infarction, uncontrolled hypertension, atrioventricular block, persistent atrial fibrillation, severe valvular heart disease, left ventricular ejection fraction less than 35%, and chronic obstructive pulmonary disease were excluded.

### Procedure

#### Coronary angiography and FFR measurement

All patients underwent elective coronary angiography to determine the coronary anatomy, stenotic artery, number of diseased vessels, and the degree of stenosis in LAD. Patients with LAD intermediate stenosis (50–70%) were included in the study; then, they underwent FFR measurement to assess the functional significance of the stenosis.

To measure the FFR a 0.014-inch pressure wire was advanced via a 6-Fr guiding catheter distal to the target coronary lesion. Continuous intravenous infusion of adenosine at a dosage of 140 g/min/kg via a forearm vein for up to 3 min was used to achieve maximum hyperemia. FFR was determined as the ratio of mean distal coronary pressure to mean aortic pressure at rest and maximal hyperemia.

Patients with FFR values ≤ 0.8 were considered to have functionally significant lesions, then they underwent therapeutic stent deployment in the specified lesion. Lesions with FFR values > 0.8 were considered functionally insignificant [13]**.**

#### Conventional resting trans thoracic echocardiography

All patients were subjected conventional transthoracic echocardiography (TTE) using a (Vivid E9, General Electric Health Care Vingmed, Milwaukee, WI, USA) machine on admission for coronary angiography procedure. Standard images were taken in the parasternal (long- and short-axis images) and apical (two-, three-, and four-chamber images) views using a 3.5–5 MHz transducer at a depth of 16 cm. The cine-loop format was used to save standard 2D and color Doppler data triggered by the QRS complex. At least three consecutive beats were used to average the data. The TTE study and chamber quantifications were carried out in accordance with the American Society of Echocardiography and the European Association of Echocardiography's principles, with measures indexed to body surface area when indicated [14].

#### 2-dimensions (2-D) speckle tracking

For offline Speckle tracking analysis, the 2D echocardiography images were obtained from LV apical 3-chamber, 4- chamber, and 2- chamber views with frame rates of 50–90 frames/s during breath-holding with stable ECG, with good endocardial delineation and avoidance of foreshortening [7]. Digital data were stored and analyzed offline using EchoPac 110.1.3, GE workstation by two certified echocardiographers with level three training who were blinded to the studied groups. In each of the three apical views, the endocardial border was manually traced in a counterclockwise fashion starting at the mitral annulus at the end-systolic frame. The software then divided the LV into six evenly spaced segments (apical, mid, and basal). After that, the operator accepts segments with good quality and rejects segments with poor quality.

The global longitudinal strain (GLS) was calculated from the average strain values of the 17 LV segments. We recorded myocardial strain values of the segments supplied by LAD according to the latest chamber quantification recommendations [14]. Recorded segments were basal anterior segment (segment no. 1), mid anterior segment (segment no. 7), apical anterior segment (segment no. 13), basal anteroseptal (segment no. 2), mid anteroseptal (segment no. 8), apical septal (segment no. 14), and apical inferior segment (segment no. 15). The least absolute value of these 7 segments was adopted as the regional PLS, and its curve was used as a reference for the calculation of PSI and the duration between AVC and regional PLS [15].

The PSI (%) was calculated using the following formula: ([maximum strain change during the cardiac cycle] − [end-systolic strain])/(maximum strain change during the cardiac cycle) × 100. The greatest (least absolute) value in the analyzed LAD distribution segment was adopted as a representative value for LAD [9].

The duration between AVC and regional PLS was calculated manually as follows: [the time between regional PLS and R-wave on ECG]—[the time between AVC and R-wave on ECG] [9].

### Statistical analysis

The Statistical Package for Social Sciences (IBM SPSS Statistics) for Windows, version 26 was used to analyze the data (IBM Corp., Armonk, N.Y., USA). The distribution of continuous numerical data was assessed using the Shapiro–Wilk test for normality. Variables which were distributed normally and summarized as mean and standard deviation SD. The correlations between two numerical variables were assessed using Pearson's correlation [correlation coefficient (r) = mild0.3, moderate 0.3–0.7; and strong: r > 0.7, regardless of sign] and group comparisons were done using the unpaired student t-test. Frequencies and percentages were used to summarize categorical variables. Pearson's Chi-square and Fisher's exact tests were used to examine the relationships between the groups. The diagnostic performance of the researched indices was evaluated using a Receiver Operating Characteristic (ROC) curve analysis. The AUC was used to measure the indices' discriminating strength (excellent: 0.9–1.0; good: 0.8–0.9; fair: 0.7–0.8; and poor: 0.6–0.7). To illustrate statistical significance, a p-value of less than 0.05 was used.

## Results

The current study included 200 patients who had functionally suspicious intermediate LAD stenosis (50–70%) by coronary angiography. The patients were divided into two groups according to their FFR values. Group I included 117 patients with functionally non-significant LAD lesions (FFR > 0.8), while group II included 83 patients with functionally significant LAD lesions (FFR ≤ 0.8). While patients with significant stenosis in vessels other than LAD were excluded from our study, non-significant stenotic lesions in vessels other than LAD was documented in 57 (48.7%) cases in group I, 35 (42.2%) cases in group II with no statistical difference between the two groups (P value = 0.36).

The baseline characteristics were comparable between the two groups (Table 1). As regards conventional echocardiographic parameter, In group I, LVEF ranged from 52 to 72%, while in group II LVEF ranged from 55 to 68% with no abnormal ejection fraction (below 52%) documented in any of the studied groups. Table 2 shows no significant differences between the two groups as regards the resting conventional echocardiographic parameters.

**Table 1:** Comparison between the studied groups regarding demographic data and clinical characteristics

Parameter	Groups		Test
	Group I (N = 117) (FFR > 0.8)	Group II (N = 83) (FFR ≤ 0.8)	P
Age (year), Mean ± SD	59.02 ± 11.64	62.0 ± 7.15	0.176
Gender Male, n (%) Female, n (%)	51 (43.6) 66 (56.4)	31 (37.3) 52 (62.7)	0.504
Diabetes mellitus, n (%)	69 (59)	56 (67.5)	0.221
Hypertension, n (%)	86 (73.5)	53 (63.9)	0.144
Dyslipidemia, n (%)	99 (84.6)	65 (78.3)	0.253
Smoking, n (%)	37 (31.6%)	22 (26.5%)	0.434
Family history of coronary artery disease, n (%)	0	2 (2.4%)	0.171

*FFR* fractional flow reserve, *SD* standard deviation

**Table 2:** Resting conventional transthoracic echocardiographic parameters between the studied groups

Parameter	Groups		Test
	Group I (N = 117) (FFR > 0.8) Mean ± SD	Group II (N = 83) (FFR ≤ 0.8) Mean ± SD	P-Value
Heart rate	82 ± 1065	84 ± 9.85	0.178
SBP	119 ± 12.25	120 ± 9.78	0.537
LV end-diastolic dimension (mm)	44.044.49	45.99 ± 4.24	0.136
LV end-systolic dimension (mm)	28.87 ± 5.69	30.35 ± 4.50	0.051
LV end-diastolic volume (ml)	80.51 ± 21.88	84.92 ± 20.13	0.148
LV end-systolic volume (ml)	33.43 ± 18.18	36.11 ± 12.31	0.244
LV ejection fraction (%)	59.97 ± 5.38	62.28 ± 4.40	0.060
LV post wall thickness (mm)	9.78 ± 1.93	9.87 ± 1.99	0.751
Interventricular septal thickness (mm)	9.82 ± 1.95	10.17 ± 1.72	0.193
Left atrial volume index (ml/m^2^)	24.53 ± 7.41	23.99 ± 6.08	0.592
Mitral valve E/A ratio	1.06 ± 0.461	1.020 ± 0.278	0.846
Mitral valve deceleration time (msec)	215.17 ± 57.48	201.64 ± 33.38	0.060
Mitral valve E/E` ratio	9.49 ± 2.62	8.91 ± 4.67	0.258

*FFR* fractional flow reserve, *SD* standard deviation, *LV* left ventricle, *SBP* systolic blood pressure

Table 3 compares the findings of STE on admission between the studied groups. Both the regional PLS and the global longitudinal strain (GLS) had significantly lower absolute values in patients with functionally significant LAD stenosis compared to those with functionally non-significant lesions (− 14.98 ± 5.05 and − 18.73 ± 3.92 vs. − 17.59 ± 3.62 and − 19.20 ± 2.61, p = 0.001 and 0.02, respectively).

**Table 3:** Comparison of speckle tracking on admission between studied groups

Parameter	Groups		Test
	Group I (N = 117) (FFR > 0.8)	Group II (N = 83) (FFR ≤ 0.8)	P
Regional PLS Mean ± SD Range	− 17.59 ± 3.62 − 21 to − 13	− 14.98 ± 5.05 − 20 to -4	0.001*
GLS Mean ± SD Range	− 19.20 ± 2.61 − 24 to − 15.5	− 18.73 ± 3.92 − 24 to − 11	0.02*

*FFR* fractional flow reserve, *SD* standard deviation, *PLS* peak longitudinal strain, *GLS* global longitudinal strain

As regard The Post systolic strain index (PSI), it was 9.93 ± 6.4 in group I vs 13.08 ± 6.78 in group II, with statistically significant difference between the studied groups (P = 0.001). While, duration between AVC & regional PLS was 50.97 ± 28.14 in group I, 53.05 ± 28.43 in group II, with statistically significant difference between the studied groups (P = 0.02).

**Fig. 1:**
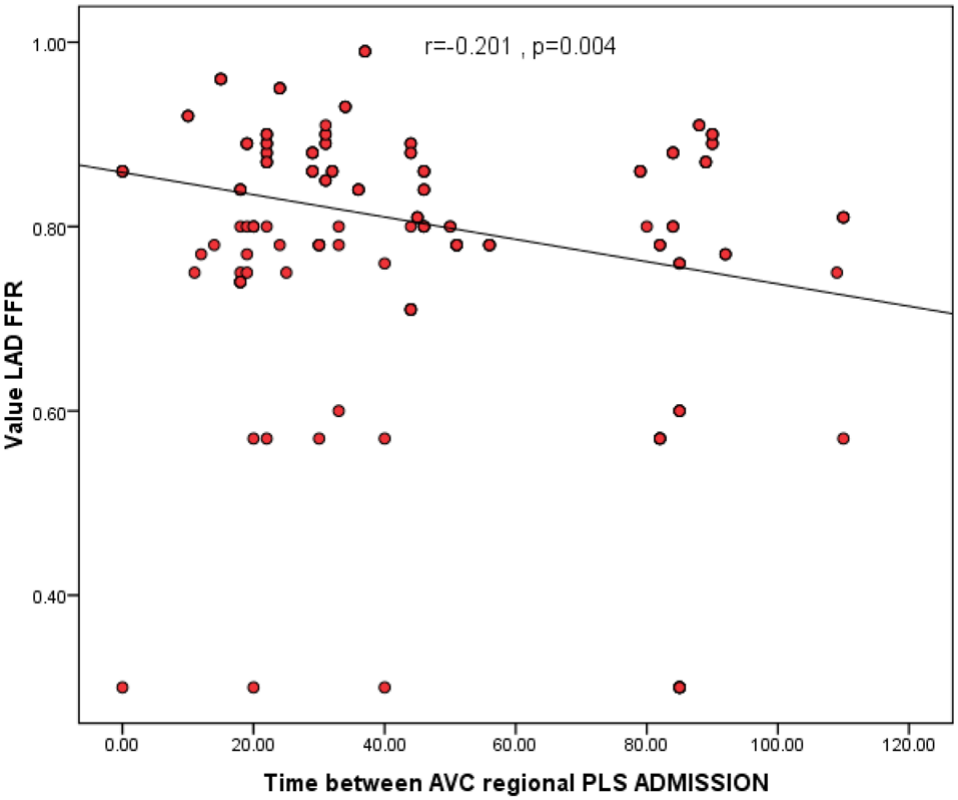
Scatter plot showing significant Correlation between the time interval between AVC & regional PLS measured using 2D speckle tracking against invasive FFR values for intermediate LAD coronary lesions. The correlation coefficient of PSI against FFR values was r = − 0.201(P = 0.004)

**Fig. 2:**
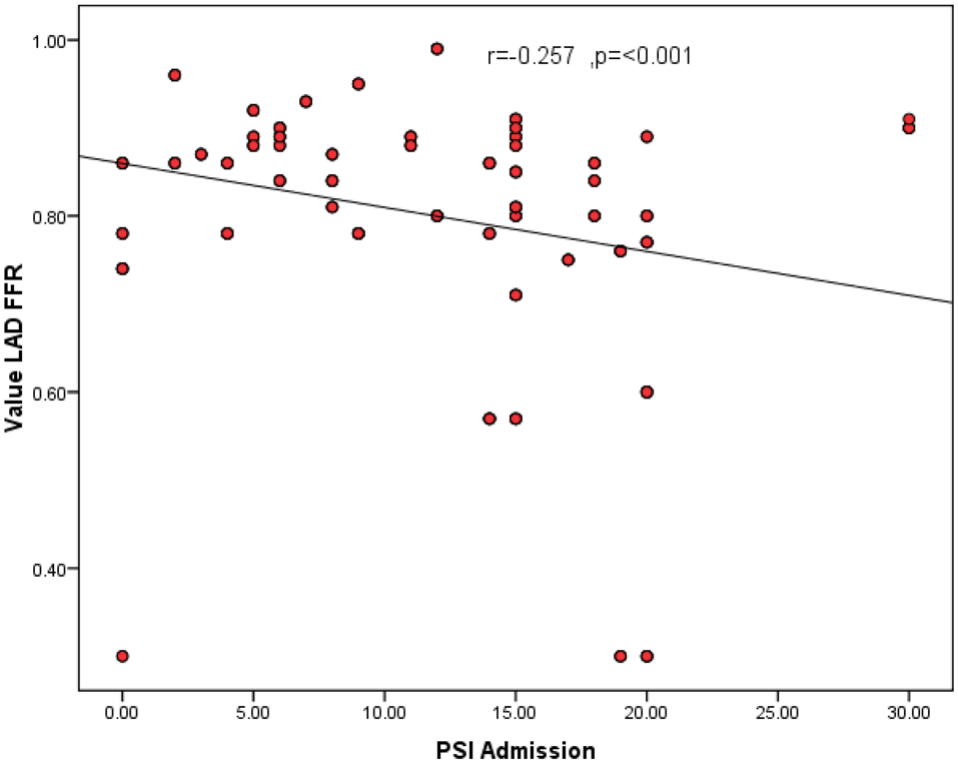
Scatter plot showing significant Correlation between PSI measured using 2D speckle tracking and invasive FFR values for intermediate LAD coronary lesions. The correlation coefficient of PSI against FFR values was r = − 0.257 (P < 0.001)

We found a significant negative correlation between the value of LAD FFR and each of the time interval between AVC and regional PLS on admission (r = − 0.201, P = 0.004, Fig. 1) and the PSI on admission (r = − 0.257, P < 0.001, Fig. 2).

**Fig. 3:**
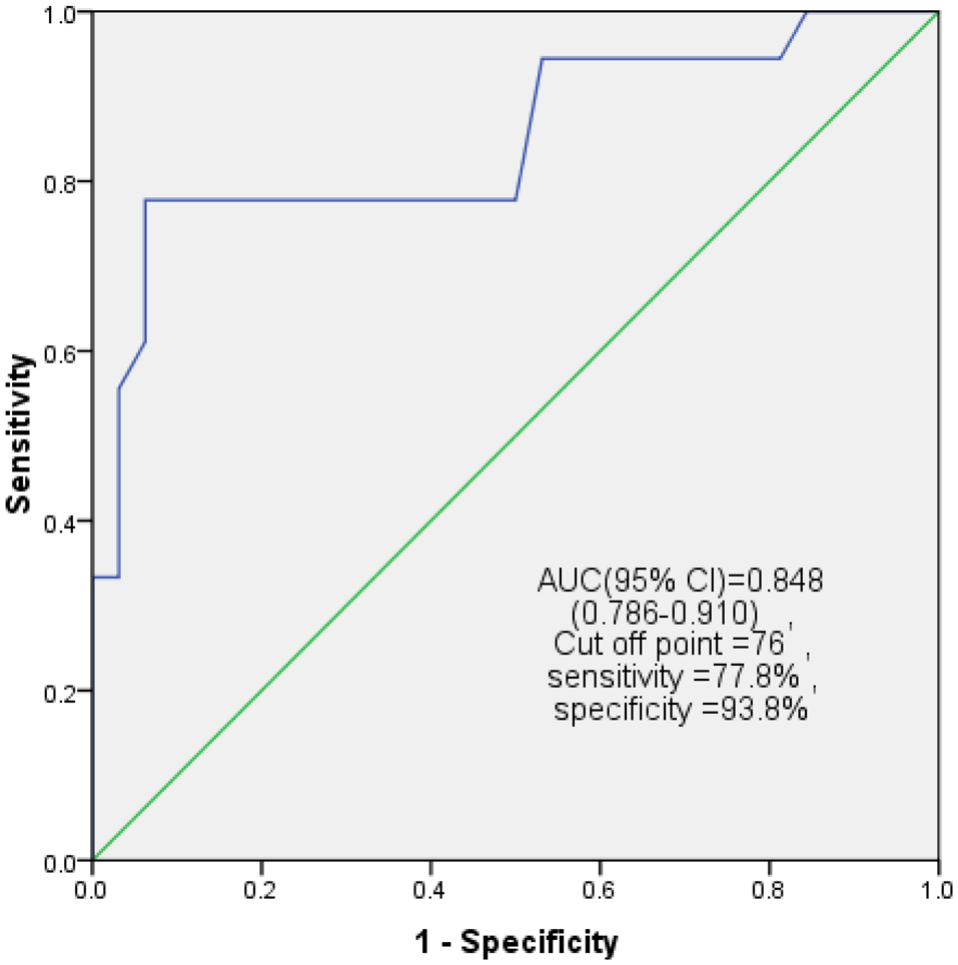
Receiver operating characteristic (ROC) curve of the time between AVC & regional PLS. Area under curve (AUC) was 0.848, with best cut-off value at 76 ms in diagnosing functionally significant intermediate LAD lesions (FFR ≤ 0.8) with 77.8% sensitivity and 93.8% specificity; CI: confidence interval

**Fig. 4:**
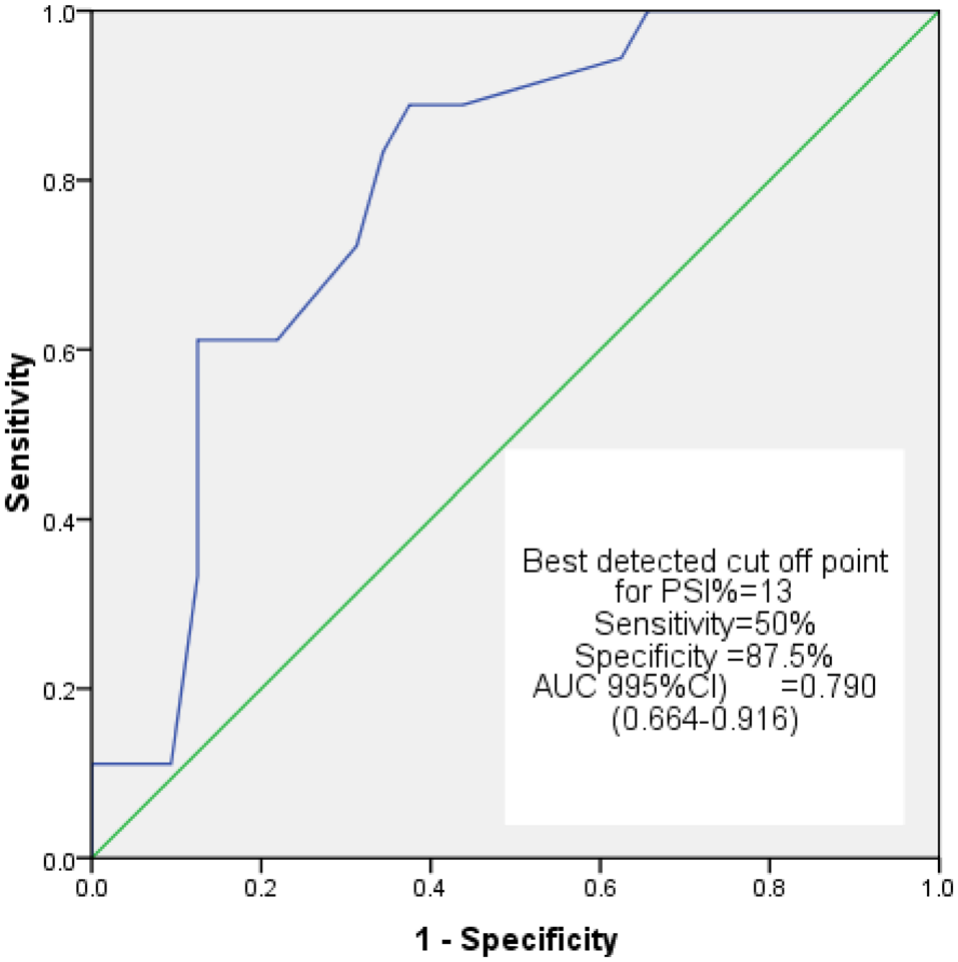
Receiver operating characteristic ROC curve of PSI.Area under curve (AUC) was 0.790, with best cut-off value at 12% in diagnosing functionally significant intermediate LAD lesions (FFR ≤ 0.8) with 50% sensitivity and 87.5% specificity; CI: confidence interval

**Fig. 5:**
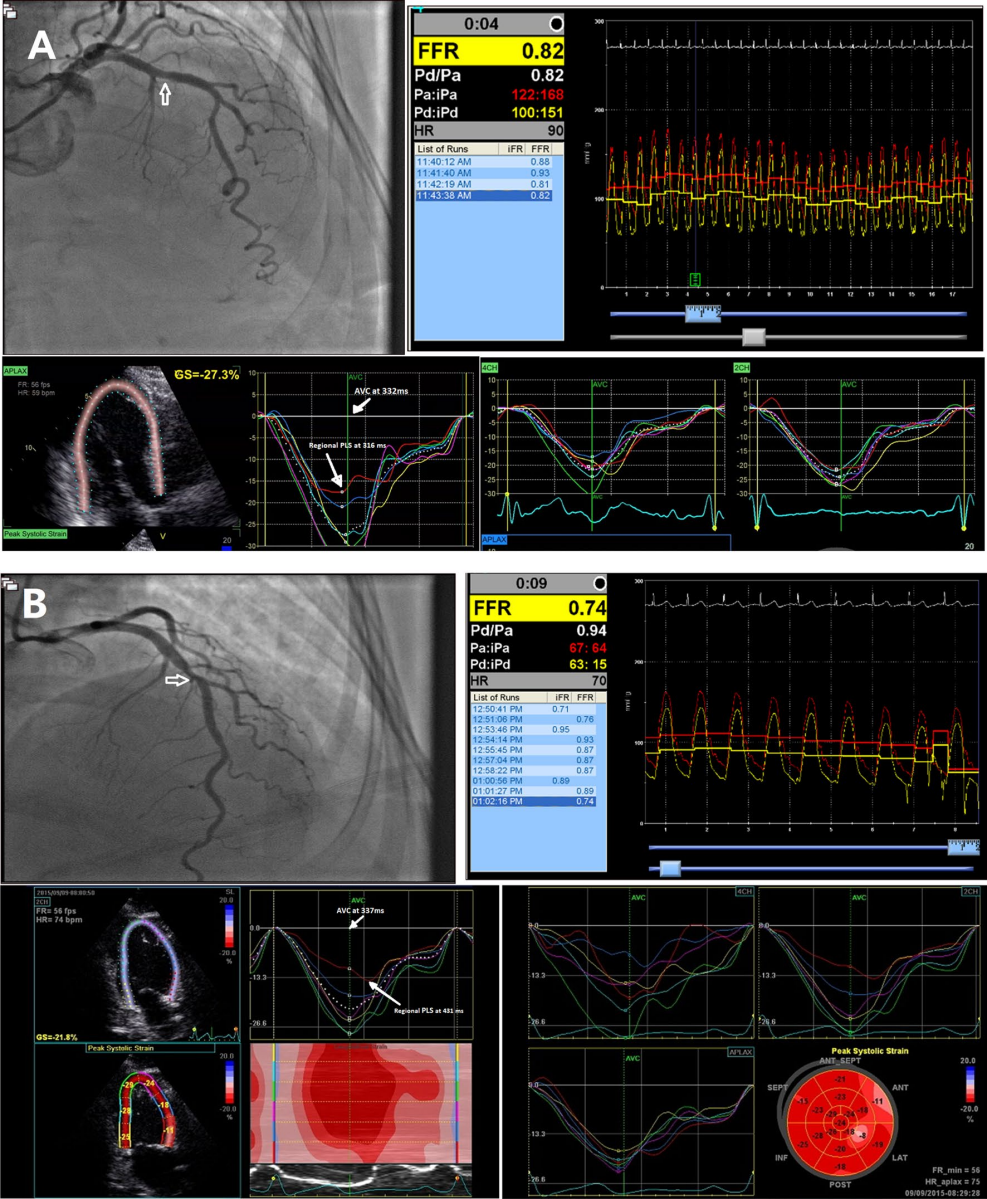
**A** Demonstrates case example from group I, diagnostic coronary angiography showed mid LAD stenotic lesion of 55%, FFR was 0.82 confirming functionally non-significant stenosis. The least PLS of segments supplied by LAD was in the basal anteroseptal segment (yellow arrow) (− 18%) representing the regional PLS. Regional PLS (red curve in apical long-axis view) was immediately before AVC (green vertical line) with no evidence of post systolic strain. Aortic valve closure was at 332 ms, peak longitudinal strain of the basal anteroseptal segment was at 316 ms. **B** Demonstrates case example from group II, diagnostic coronary angiography showed intermediate mid LAD stenotic lesion of 60%, FFR was 0.74 confirming functionally significant stenosis. The least PLS of segments supplied by LAD was in the basal anterior segment (yellow arrow) (− 11%) representing the regional PLS. AVC (green vertical line) was at 337 ms and regional PLS (red curve at apical 2 chamber view) was at 431 ms, time between AVC and regional PLS was calculated at 431–337 = 94 ms

The results of the ROC curve analysis revealed that the interval between AVC and regional PLS had good discriminatory power (AUC 0.848, 95% CI 0.786–0.910) and the best-detected cut-off value was 76 ms, with 77.8% sensitivity and 93.8% specificity (Fig. 3). As for PSI, the AUC showed fair to good discriminatory power (AUC 0.790, 95% CI 0.664–0.916), and the best cut-off value was 13%, yielding 50% sensitivity and 87.5% specificity (Figs. 4, Example from studied cases was demonstrated in (Fig. 5) showing no delay between AVC and regional PLS in patient with FFR > 0.8 while in patient with FFR.

## Discussion

Assessment of the functional significance of intermediate coronary lesions is challenging; however, it is crucial for clinical decision-making. Invasive FFR was considered the gold standard to differentiate between functionally significant and non-significant lesions using a cut-off value of 0.8 [3, 16–18].

The resting TTE is a rapid, non-invasive test compared to the gold standard FFR which is an invasive procedure that requires a hospital stay and carries a risk of radiation and contrast exposure [19, 20]. The STE used to measure myocardial deformation indices is known to be a semi-automated, sensitive, and reproducible tool for the assessment of myocardial ischemic segments [21].

Few studies investigated the relationship between the functional significance of intermediate LAD coronary stenosis measured by invasive FFR in stable CAD patients and the regional LS. Therefore, the present study aimed to assess the diagnostic accuracy of resting myocardial deformation indices in detecting the functional significance of intermediate LAD coronary artery lesions. To the best of the authors’ knowledge, this is the first study with an adequate sample size to investigate the correlation between FFR values and PSS parameters including PSI and the duration between AVC and regional PLS obtained from the territory of LV segments supplied by LAD coronary artery.

In the current study, the resting conventional TEE parameters revealed no significant differences between the studied groups. On the contrary, the regional PLS and GLS showed significantly lower absolute values in patients with FFR ≤ 0.8 than those with FFR values > 0.8. This finding supports the hypothesis that myocardial strain is a more sensitive alternative to standard echocardiographic parameters, which are not always sensitive enough to detect subtle changes in myocardial function [22]. Also, strain reflects the contractile function of the subendocardial fibers, which are predominantly affected by ischemia [23].

These findings are supported by the results of previous studies. Gaibazzi et al. [24] investigated the resting GLS ability to detect significant CAD in 82 patients with > 50% coronary artery stenosis undergoing stress echocardiography and coronary angiography. The researchers found that the patients with significant CAD had significantly reduced rest GLS, but not LVEF or end-diastolic volume, compared to those with non-obstructive CAD.

Dobrowolski et al. [15] evaluated 30 patients with significant LAD stenosis confirmed by FFR values ≤ 0.8. The study compared anterior and inferior walls in the two-chamber apical view in the same group of patients. The researchers reported significantly lower absolute values of longitudinal systolic strain in the anterior wall segments compared to the inferior wall segments in the same group of patients.

Nishi et al. [25] examined three-dimensional myocardial longitudinal strain indices on resting STE in patients with stable CAD confirmed by FFR. The investigators documented that the longitudinal strain values were significantly lower in segments supplied by functionally stenotic vessels with FFR ≤ 0.8 compared to those supplied by the non-functionally stenotic vessels.

Xing and colleagues [26] used two-dimensional STE to assess left ventricular systolic performance in individuals with various types of ischemic heart disease. They found no significant differences between the studied groups regarding the conventional echocardiographic parameters. However, the group of patients with functionally significant CAD, either obstructive or coronary microvascular diseases, had significantly lower absolute values of global and regional longitudinal strain compared to the control group.

The present study also investigated the PSI and the duration between AVC and PLS as parameters for the assessment of PSS. The PSS was proposed as an ischemia marker and was linked to a degree of coronary flow reduction. The PSS was also found to be negatively correlated with measures of LV systolic function in ischemic areas [27]. The ROC curve analysis demonstrated that the PSI enabled us to identify the ischemic segments supplied by a functionally significant stenotic artery using a cut-off point of 13%, yielding 50% sensitivity and 87.5% specificity). Furthermore, the duration between AVC and regional PLS at a cut-off point of 76 ms differentiated between segments supplied with functionally significant and non-significant LAD stenosis with 77.8% sensitivity and 93.8% specificity.

The diagnostic utility of PSS measures to detect the ischemic segments supplied by stenotic LAD lesions with an FFR value ≤ 0. 8 was evaluated in a prior study by Ozawa et al. [9]. The researchers recorded the resting multi-layer 2D STE measures to detect ischemic segments confirmed by FFR in 39 patients with 46 coronary lesions. They described cut-off values of 12% for PSI and 88 ms for the duration between AVC and regional PLS. Although the AUC in their study was not statistically significant, this may be explained by the low number of the studied patients (39 patients) compared to the current study (200 patients).

Our findings indicate that the thresholds of PSI as well as the duration between AVC and regional PLS, as measures of PSS, can help the detection of LV myocardial ischemic segments in functionally significant intermediate LAD lesions.

Moreover, the cut-off points for the detection of the myocardial ischemic segments can be used as adjunctive to the standard diagnostic tools to differentiate functionally significant from non-functionally significant intermediate LAD lesions in patients with stable CAD.

Although this study was powered to test the diagnostic performance of the tested measures using ROC curve analysis, the results may be limited by non-testing of the agreement between the echocardiographic operators. In addition, stress echocardiography was not performed in the current study. Further studies need to be conducted using stress echocardiography to reveal the effect of exercise or medications on the same parameters for the detection of ischemic segments confirmed by FFR.

### Study limitations

The current study was single centered. Although this study was powered to test the diagnostic performance of the tested measures using ROC curve analysis, the results may be limited by non-testing of the agreement between the echocardiographic operators. In addition, stress echocardiography was not performed in the current study, using stress echocardiography may reveal the effect of exercise or medications on the same parameters for the detection of ischemic segments confirmed by FFR.

### Clinical implication

Resting myocardial deformation indices including regional PLS and GLS can be used as non-invasive tool to aid in assessment of intermediate coronary artery stenosis. Also, using the appropriate cut-off values of PSI and duration between AVC and regional PLS can identify functionally significant LAD stenotic lesions.

## Conclusion

In patients with stable CAD having 50–70% LAD coronary artery stenotic lesions, the 2-D speckle tracking regional PLS and GLS had significant correlations with FFR values. The PSS measures, including PSI and the duration between AVC and regional PLS, enabled the identification of segments with FFR values ≤ 0. 8 using the appropriate threshold with reasonable diagnostic accuracy.

### Recommendation

Future studies can test significance of measures on larger cohort. As well as, further studies can be conducted using stress echocardiography to reveal the effect of exercise or medications on the same parameters for the detection of ischemic segments confirmed by FFR.

## Data Availability

Our case–control study data used to support the findings of this study are available from the corresponding author upon request.

## Abbreviations

**2-D**: 2-Dimensional
**AUC**: Area under curve
**AVC**: Aortic valve closure
**CAD**: Coronary artery disease
**CI**: Confidence interval
**ECG**: Electrocardiogram
**EF**: Ejection fraction
**FFR**: Fractional flow reserve
**Fr**: French
**GLS**: Global longitudinal strain
**IRB**: Institutional Review Board
**LAD**: Left anterior descending
**LS**: Longitudinal strain
**LV**: Left ventricular
**LVEF**: Left ventricular ejection fraction
**ms**: Millisecond
**PLS**: Peak longitudinal strain
**PSI**: Post-systolic strain index
**PSS**: Post systolic shortening
**ROC**: Receiver operating characteristic
**SBP**: Systolic blood pressure
**SD**: Standard deviation
**STE**: Speckle tracking echocardiography
**TTE**: Transthoracic echocardiography
